# Salvage therapy with *Sodium chlorosum* (formerly DAC N-055) for cases of refractory lupoid cutaneous leishmaniasis: results from a compassionate use study with 0.09% *Sodium chlorosum* in amphiphilic basic cream

**DOI:** 10.1186/s12879-019-4518-x

**Published:** 2019-11-28

**Authors:** Sara Molkara, Elaheh Poursoltani, Kurt-Wilhelm Stahl, Masoud Maleki, Ali Khamesipour, Christian Bogdan, Maryam Salehi, Vahid Mashayekhi Goyonlo

**Affiliations:** 1 Cutaneous Leishmaniasis Research Center, Mashhad University of Medical Sciences, Mashhad, Iran; 2 Waisenmedizin e. V. Promoting Access to Care with Essential Medicine (PACEM), Non-Profit Non-Governmental Organization, Freiburg, Germany; 3 Center for Research and Training in Skin Diseases and Leprosy, Tehran University of Medical Sciences, Tehran, Iran; 4 Mikrobiologisches Institut – Klinische Mikrobiologie, Immunologie und Hygiene, Friedrich-Alexander-Universität (FAU) Erlangen-Nürnberg und Universitätsklinikum Erlangen, Erlangen, Germany; 5 Community Medicine Department, Faculty of Medicine and Clinical Research Unit, Ghaem Hospital, Mashhad University of Medical Sciences, Mashhad, Iran

**Keywords:** Lupoid cutaneous leishmaniasis, *Sodium chlorosum* (formerly DAC N-055), Treatment

## Abstract

**Background:**

Lupoid cutaneous leishmaniasis (LCL) is known as a rare but serious complication of anthroponotic cutaneous leishmaniasis (ACL) resistant to conventional treatments. *Sodium chlorosum,* a pro-oxidative preparation of pharmaceutical sodium chlorite (NaClO_2_), has been successfully used for the treatment of Old World cutaneous leishmaniasis lesions (OWCL) and of some LCL cases in Afghanistan. This clinical trial study aimed to evaluate the effect of a last resort therapy with topical 0.09% *sodium chlorosum* on LCL in Iran.

**Methods:**

Twenty Iranian patients (12 women and 8 men) with LCL refractory to treatment were included in this salvage study. A magistral preparation of *sodium chlorosum* (10 mM NaClO_2_ in amphiphilic basic cream) was applied twice daily to the lesions for 6 weeks and continued up to 12 weeks in patients who showed a clinical response within the first 6 weeks. Responders were followed up for a maximum of 1 year. Lesions were photographed during weekly visits. Disappearance of erythema and indurated lesions were rated as complete clinical response.

**Results:**

Patients with a mean age of 28.6 (±24.3) and with an ACL proven lesion history of 3.8 (±1.4) years were treated for an average of 7.9 (±1.8) weeks. At the end of the treatment period (12th week), a complete response was observed in 9 of 20 patients (45%). During the one-year follow-up period, LCL lesions recurred in 4 of these 9 patients (with one patient showing only a tiny lesion) and one case lost to follow up whereas the other four remained completely lesion-free. Mild temporary side-effects such as erythema and itching were seen in 4 of 20 patients (20%).

**Conclusions:**

Topical *sodium chlorosum* showed promising therapeutic results and can be considered as safe, painless, and relatively effective treatment for LCL, an ethical prerequisite for a two-armed controlled trial.

**Trial registration:**

This study was registered in Iranian registry of clinical trials on 2019-02-02 with registration number IRCT20190114042356N1.

## Background

Cutaneous leishmaniasis (CL) is a vector-borne infective disease of the skin [1]. Over two thirds of new CL cases occur in only six countries: Afghanistan, Algeria, Brazil, Colombia, Iran, and Syria. In Iran, the Khorasan province and particularly Mashhad city is a major endemic area for anthroponotic CL (ACL) caused by *L. tropica* [2].

4–10% of ACL cases in Iran and Afghanistan develop non-healing lupoid forms of CL. Chronic lesions of lupoid leishmaniasis (LCL) present as red brown papules and plaques around or at the site of primary lesion [3]. Between 2012 and 2013, 3857 ACL patients were registered in Herat province, Afghanistan, at a 200 miles flight distance from Mashhad. 4.2% of them presented Giemsa smear negative, PCR-positive lesions of lupoid CL caused by *L. tropica* with a lesion history of 2 to 5 years. 85.8% of them were aged < 20 years, i. e. the major part of their life still lying ahead of them with such debilitating skin lesions. This showed that LCL, although rare, constitutes a heavy disease burden in some areas [4].

Despite the activation of cellular immunity, LCL is associated with an ineffective Th1 response that is unable to properly resolve the lesion leading to chronicity of disease [5, 6]. The role of T cells in pathogenesis of LCL lesions is complicated and poorly understood. The conventional Th1/Th2 concept can neither readily explain the improvement nor the progression of disease. However, recent studies suggest that a type IV hypersensitivity reaction and/or regulatory T cells and IL-17 prevail in LCL patients [6, 7].

To date, there is no clinically proven effective treatment strategy for LCL. There are, however, some case reports dealing with cryotherapy, thermotherapy, ablative and non-ablative lasers, local and systemic antimony compounds, amphotericin B, oral antibiotics, and anti-fungal agents which have all been used for treatment of LCL. But LCL lesions are usually resistant to these treatments and persist or extend slowly for months and years [8, 9].

Clinical evidence suggests that a special preparation of sodium chlorite for pharmaceutical use (*sodium chlorosum*), which is produced in such a way that it contains less than one mole percent of toxic chlorate, can promote the healing of different types of chronic wounds [10], including CL wounds [11, 12]. In 1983, *sodium chlorosum* was registered in Germany as a finished wound healing chlorite solution supposedly containing tetradecachlorooxygen (Cl_4_O_10_, TCDO). *Sodium chlorosum* was later monographed as DAC N-055 in the German Drug Codex from 1989 to 2014. *Sodium chlorosum* is now a component of the filmogenic LeiProtect® lesion dressing gel which in August 2017 has received a special approval as medicinal device by the German Federal Institute for Drugs and Medical Devices (BfArM) (K.W. Stahl, personal communication, BfArM license no. 91.1.07-5640-S-006/16).

Some 30 years ago, the former “TCDO” has been shown to promote cellular proliferation, the function of immune cells and hematopoiesis [13–16], an effect, which NaClO_2_, commercialized as a chemical compound, did not have in rodents [17].

After some case reports describing the remarkable effects of topical application of *sodium chlorosum* (0.045 to 0.09% in different formulations) on LCL lesions [18, 19], the present study aimed to assess the LCL salvage effects of topical 0.09% *sodium chlorosum* (formerly DAC N-055) containing 10 mM NaClO_2_ with < 1 Mol% NaClO_3_ in amphiphilic basic cream (DAC B-022) in 20 LCL patients.

## Method

This clinical trial study was performed as a one-armed salvage study in Imam Reza Hospital, Mashhad, Iran, with 20 LCL patients from February 2014 to February 2015. All 20 included patients originated from Razavi-Khorasan (North East of Iran), had lesions clinically compatible with LCL, were diagnosed with a proven CL history based on positive parasitological smear or histopathologic examination, and had shown resistance to previous treatment. Previous CL treatment comprised: Six patients previously treated with intra-lesional glucantime injection for at least 12 weeks, five with intra-lesional Glucantime® plus cryotherapy, four with systemic Glucantime® for at least 20 days, four patients who underwent treatment with intra-lesion Glucantime® plus amphotericin-B, and one with intra-lesional Glucantime® plus topical diphenylcyclopropenone (diphencyprone), an immunostimulatory drug approved in China and New Zealand, but not in Europe or the United States, nor in Iran.

Patients, who had been treated for CL during the previous 2 months or were pregnant or lactating, were excluded. Study strategy and interventions were fully explained to patients and informed written consent was obtained from patients and/or parents. This clinical trial was registered in the Iranian registry of clinical trials (IRCT) with the registration code IRCT20190114042356N1.

A magistral prescription preparation of 0.09% (10 mM) *sodium chlorosum* in DAC B-020 basic cream (adjusted to pH 8 with lactic acid), which is legal in Germany according to §21, section 2, sentence 1 of the German Medicines Act (AMG) to treat individual cases, was procured by the non-profit German NGO Waisenmedizin (WM e. V. – PACEM Promoting Access to Cure with Essential Medicine, www.waisenmedizin.org). WM e. V. was legally responsible for the GMP preparation of a stock solution of *sodium chlorosum* containing 1 M NaClO_2_, which is typically produced preventing the formation of chlorate. The patients were instructed to administer the amphiphilic cream topically themselves on their LCL lesions with a semi-occlusive nylon bandage twice a day for at least 6 weeks at home. The patients were clinically assessed once per week and photographs were taken of their lesions in our leishmania consultation center.

Due to the chronic and stable behavior of LCL lesions that is not supposed to change in few months, the assessment of efficacy was pre and post treatment comparison. If patients showed no improvement (induration and erythema reduction) after the first 6 weeks (PHASE I), they were considered as non-responders and their PHASE II follow-up was discontinued. However, if the patients were compliant and eager to continue the treatment, it was continued for a further period of 6 weeks until the patients terminated the treatment. In such a way, we took into account the patients’ attitude to our salvage trial, as has been recently recommended by Erber and co-authors [20]. If patients showed signs of improvement within the first 6 weeks with a significant reduction of induration and erythema, their treatment was continued for up to 6 more weeks (PHASE II).

Patients with an almost complete resolution of erythema and induration during PHASE II were considered as complete responders.

These responders were monitored for one more year during PHASE III. Absence of any erythema and induration after 12 months was rated as complete clinical response. Recurrence of any inflammation and induration after a one-year follow-up period was defined as incomplete response or treatment failure.

The patients were also observed for systemic and local side effects. In case of any major complications, the treatment was discontinued.

Data were entered into SPSS version 16 and the analysis was performed using Chi-square test or independent samples T-test when appropriate. *P* values of less than 0.05 were considered as statistically significant.

## Results

Of the 20 patients, 12 (60%) patients were women and 8 (40%) were men. With a mean age of 28.6 ± 24.3 years, and the mean duration of LCL lesions was 3.8 ± 1.4 years, similarly to what was recently observed in Herat [4]. There was no significant difference between the demographic characteristics of cured patients (i.e. those with a complete response) and non-cured ones (i.e. those with an incomplete response) (Table 1).

**Table 1:** Demographic and medical information of patients

	Cured	Non-cured	*P*
Age	24.5 ± 22.5	33.7 ± 26.8	0.407^*^
Sex			
Female	5 (41.7)	7 (58.3)	0.714^**^
Male	4 (50)	4 (50)	
Location			
Head and neck	8 (50)	8 (50)	0.592^**^
Other parts	1 (33.3)	2 (66.7)	
Disease duration (months)	38.6 ± 35.8	45.6 ± 43.2	0.706^*^
Treatment duration (weeks)	7.09 ± 1.2	9 ± 2	0.019^*^

^*^Independent samples T-test was used

^**^Chi-square test was used

All 20 patients had been refractory to previous conventional CL treatment (see Method section). They were treated with 0.09% *sodium chlorosum* in basic cream for an average duration of 7.9 ± 1.8 weeks. Table 2 shows the location of lesions and response rate in the patients. After 12 weeks, 9 out of 20 patients (45%) showed complete response (no erythema or induration), which indicates an initial cure rate of 45%.

**Table 2:** Location of lesions and response rate

Code	Location	Treatment duration (weeks)	Treatment response: reduction of erythema and induration after 12 weeks (Phase I)	Recurrence in 3 months (Phase II)	Recurrence in 12 months (Phase III)
1	Head and neck	8	incomplete	NA	NA
2	Head and neck	9	incomplete	NA	NA
3	Head and neck	7	complete	No	Yes^a^
4	Upper limb	9	incomplete	NA	NA
5	Head and neck	7	complete	No	Yes
6	Head and neck	10	complete	No	No
7	Head and neck	7	complete	Yes	NA
8	Head and neck	6	incomplete	NA	NA
9	Head and neck	6	incomplete	NA	NA
10	Head and neck	8	incomplete	NA	NA
11	Head and neck	11	complete	No	No
12	Head and neck	12	complete	No	Dropout
13	Head and neck	7	incomplete	NA	NA
14	Upper limb	11	complete	No	No
15	Head and neck	7	complete	Yes	NA
16	Head and neck	9	complete	No	No
17	Head and neck	6	incomplete	NA	NA
18	Upper limb	6	incomplete	NA	NA
19	Head and neck	6	incomplete	NA	NA
20	Upper limb	7	incomplete	NA	NA

*NA* Phase Inot analyzed

^a^a tiny erythematous plaque had remained

**Fig. 1:**
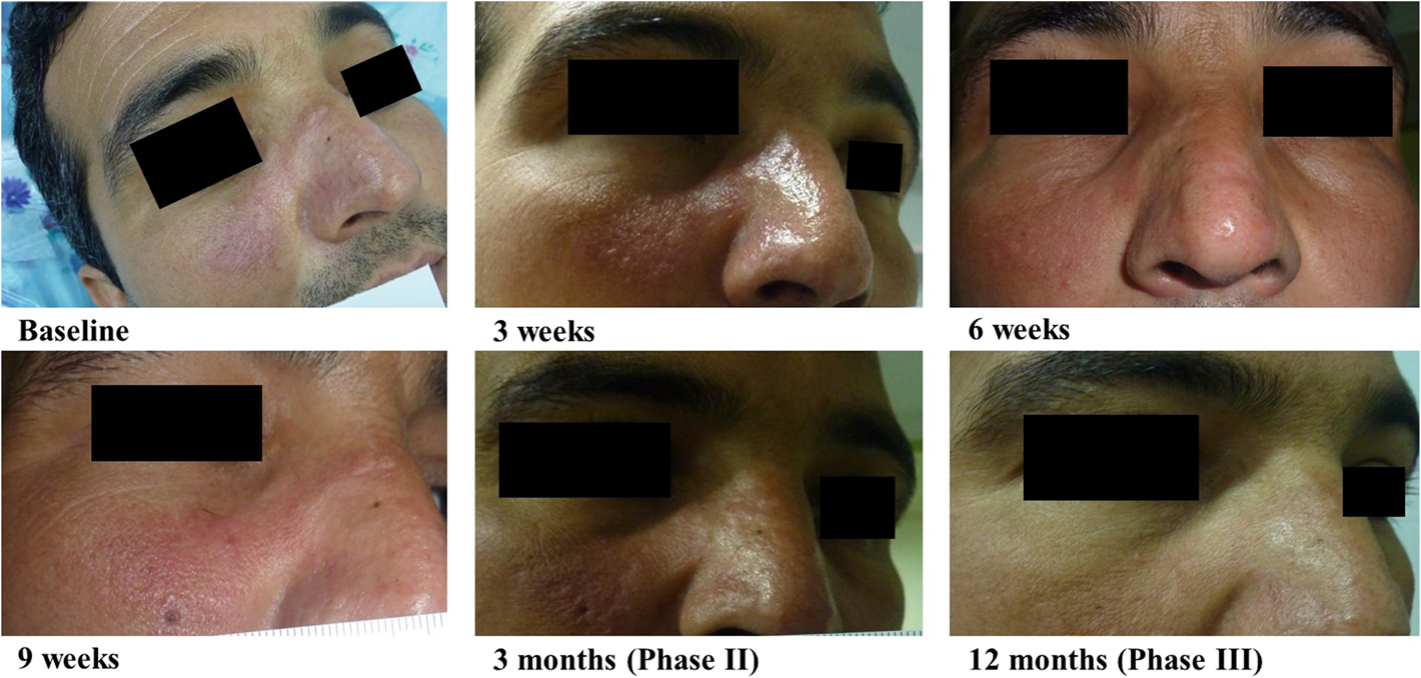
Serial photographs of a patient with complete response to the treatment showing the course of healing in three phases of the study

In two of these 9 primary responders prominent signs of recurrence were observed after 3 months (PHASE II) (see Table 2). Furthermore, two other patients showed signs of recurrence during PHASE III (12 months of follow-up), with one showing only a tiny erythema and induration at the site of lesion. Besides, 1 out of 9 primary responder patients had dropped out (lost to follow-up). The remaining four patients remained completely lesion-free. The lesion development of one these four patients is shown in Fig. 1. Overall, the primary cure rate (complete healing) after 3 months was 45% and in a one-year follow-up period, 4/20 patients remained cured which denotes a final cure rate of 20%. Regarding the adverse events, 4 (20%) of all 20 included patients reported local transient erythema and itching which were slight, did not necessitate discontinuation of treatment.

## Discussion

LCL is a rare complication of CL. LCL has a treatment-resistant nature, with most therapeutic strategies showing a slow and low response rate for this form of CL. In the present study, we assessed the therapeutic effect of a topical monotherapy of LCL with 0.09% *sodium chlorosum* in basic cream, which is neither an invasive nor a painful treatment. However, the effect is not an instant one, but requires patience on the patients’ side. We found that 9 of 20 patients (45%) showed complete response after 7 to 12 weeks of treatment. Two of these 9 cases with primary improvement suffered a recurrence at the 6th month of follow-up. After a one-year follow-up, four of the remaining 7 cases still remained cured while two of these patients relapsed (one with only a mild lesion) and the third patient had dropped out after 12 months.

All four relapses, two of them after 3 and 2 of them after 12 months, concerned patients who had followed treatment for 7 weeks only.

*Sodium chlorosum* is a pro-oxidant which after activation by hemoproteins yields a compound I oxidant with a medium redox activity [21]. As such the inorganic chlorine-oxygen pro-oxidant has been shown to improve wound healing in vivo [10–12], to exert anti-parasitic effects in vitro [11] and to have immunomodulatory properties in vitro [13]. The present findings confirm for the first time the therapeutic benefit of topical 0.09% *sodium chlorosum* in LCL patients, which was previously seen in a few cases in Afghanistan [17, 18]. In the present study, we have analyzed 20 LCL patients and succeeded in monitoring 9 of the 20 initially included LCL patients for 1 year. In the current salvage trial on a rare form of CL, the overall number of patients was too small to assess the impact of factors such as age, sex and the lesion site on the healing of LCL lesions with topical *sodium chlorosum* cream in a meaningful way. Retrospectively, it might have been a mistake to categorize the 11 patients that did not show improvement of their skin lesions after 6 weeks of initial treatment as permanent non-responders and to discontinue the application of topical *sodium chlorosum*. Therefore, advising the patients properly to be more compliant and have more patience in the course of treatment until a favorable outcome is reached should be recommended. Consequently, these salvage trial results may pave the way for an ethical two-armed comparative trial with the *sodium chlorosum* gel LeiProtect® as a finished product, which has been specially approved by the German health authorities. In any case, *sodium chlorosum* formulations should be applied for longer treatment periods than in the present trial.

The present topical magistral application of *sodium chlorosum* in amphiphilic basic cream using a nylon bandage can be considered as semi-occlusive dressing. A completely occlusive dressing could eventually have further enhanced the penetration of *sodium chlorosum* through the mostly intact skin barrier of LCL lesions.

In an earlier Iranian study, a triple therapy with cryotherapy, paromomycin, and glucantime was used to treat lupoid and chronic LCL. This treatment initially cured 65.3% of 23 patients after 10 weeks [22]. However, it is far more complicated to administer than the topical treatment used in this trial; due to the ease of use of our treatment, a 45% improvement in 12 weeks is comparable to the results of their study. The difference in the recurrence rate between this previous study (1/23 patients) by Nilfrousihzadeh et al. [22] and the present study might be related to the shorter follow-up period in the earlier analysis (3 months [22] vs. 12 months here).

In a case series with five LCL patients in Northern Afghanistan, 2/5 patients (40%), who were successfully treated with 0.045% DAC N-055, were followed up for 26 months and showed no relapse within this observation period [19].

The preparation formerly named TCDO*,* which was produced in a similar way as *sodium chlorosum,* has been shown to activate the phagocytic capacity of macrophages resulting in improved wound healing in humans [10] and to stimulate natural killer (NK) cells in rodents [17]. The importance of macrophages and dendritic cells in skin lesions of CL has been shown by Taheri et al. [23]. Monocytes, macrophages, and dendritic cells are critical for the activation of NK cells in mouse and human leishmaniasis [24, 25]. The effect of this drug on the healing of cutaneous leishmaniasis in our study may be related to its effect on macrophage activity.

## Conclusion

Our findings show that topical 0.09% *sodium chlorosum* treatment can be considered as a non-invasive, safe, and non-painful monotherapy to treat resistant and non-healing lesions of LCL.

Considering the treatment-resistant, progressive, and destructive character of LCL, the primary cure rate of 45% and the final cure rate of 20% after 1 year is encouraging, but may be improved by longer topical treatment periods. Topical *sodium chlorosum* formulations which have been approved or certified by health authorities should be given a fair chance to demonstrate their practicability and economic usefulness in a double-armed controlled trial versus more invasive, painful, and expensive therapeutic strategies.

## Data Availability

The datasets used and/or analyzed during the current study are available from the corresponding author on reasonable request.

## Abbreviations

ACL: Anthroponotic cutaneous leishmaniasis
CL: Cutaneous leishmaniasis
Diphencyprone: Diphenylcyclopropenone
LCL: Lupoid cutaneous leishmaniasis
NaClO_2_: *Sodium chlorosum*
NK: Natural killer
Sb(V): Pentavalent antimony (e.g. Glucantime)
TCDO: Tetradecachlorooxygen (Cl_4_O_10_)
